# Quantitative analysis of phantom studies of ^111^In and ^68^Ga imaging of neuroendocrine tumours

**DOI:** 10.1186/s40658-018-0204-0

**Published:** 2018-02-20

**Authors:** L. Jönsson, A. Stenvall, E. Mattsson, E. Larsson, A. Sundlöv, T. Ohlsson, C. Hindorf

**Affiliations:** 1 Department of Medical Radiation Physics, Clinical Sciences, Lund University, Lund, Sweden; 2 Department of Hospital Physics, County Hospital Ryhov, Jönköping, Sweden; 3 Department of Radiation Physics, Skåne University Hospital, Lund, Sweden; 4 Department of Oncology and Pathology, Clinical Sciences Lund University, Lund, Sweden

**Keywords:** Quantification, PET/CT, ^68^Ga, SPECT/CT, ^111^In, NEMA image quality phantom

## Abstract

**Background:**

Nuclear medicine imaging of neuroendocrine tumours is performed either by SPECT/CT imaging, using ^111^In-octreotide or by PET/CT imaging using ^68^Ga-radiolabelled somatostatin analogs. These imaging techniques will give different image quality and different detection thresholds for tumours, depending on size and activity uptake. The aim was to evaluate the image quality for ^111^In-SPECT and ^68^Ga-PET imaging, i.e. the smallest volume possible to visualize for different source-to-background activity ratios. The accuracy of quantification of lesion volume and activity was also investigated to develop an objective evaluation for radionuclide therapy eligibility.

The phantom study was performed using the NEMA IEC Body Phantom with six hot spheres having inner diameters of 10, 13, 17, 22, 28, and 37 mm, filled with either ^68^Ga or ^111^In with sphere-to-background ratios (SBRs) of no background activity, 5:1, 2.5:1, and 1.25:1. Activity ratios of 1.25:1 and 2.5:1 are clinically found for lesions close to the liver and spleen. Clinical acquisition and reconstruction protocols were applied. Line profiles were drawn to evaluate the smallest detectable volume within a given SBR. Recovery curves based on threshold-based VOIs, threshold-based VOIs adapted to the background and CT-based ROIs were obtained for all SBRs and sphere diameters, allowing for quantification.

**Results:**

The 10-mm sphere was not possible to detect in SPECT images. It was detectable in PET images for SBRs of 2.5:1 and higher. In a background corresponding to the activity uptake in the liver, spheres larger than 22–37 mm were detectable in the ^111^In-SPECT images and spheres larger than 13–22 mm were detectable in the ^68^Ga-PET images. The maximum activity concentration was accurately quantified for spheres larger than 22 mm in the PET images; however, the quantification was impaired by sphere size and background activity.

**Conclusions:**

It was not possible to detect the 10-mm sphere in any of the SPECT images. In a background corresponding to the activity uptake in the liver, spheres larger than approximately 30 mm were visible in the ^111^In-SPECT images and spheres larger than approximately 17 mm were visible in the ^68^Ga-PET images. Sphere diameter and background activity strongly affect the possibility of a correct quantification.

## Background

Functional imaging has become an integral part of oncological practice, useful during the diagnostic phase, and for staging and treatment evaluation. ^18^F-FDG PET is now a widely used type of functional imaging in cancer. However, for neuroendocrine tumours (NETs), it has proven less useful due to the often very low proliferative activity in these tumours, leading to a low glucose metabolism.

In the context of NETs, the first step in functional imaging came with somatostatin receptor (SSTR) scintigraphy which allowed visualization of the degree of SSTR expression in the tumours. In recent years, SSTR imaging has been further refined by using positron emission tomography (PET) instead of scintigraphy, giving a higher spatial resolution and a higher sensitivity for tumour detection. SSTR imaging is achieved by labelling a somatostatin analog with either a gamma-emitting or a positron-emitting isotope for scintigraphy and PET, respectively. More specifically, it is performed using ^111^In-octreotide (Octreoscan™) with SPECT/CT imaging or ^68^Ga-DOTA-conjugated peptides (DOTA-TATE, DOTA-TOC, and DOTA-NOC) with PET/CT imaging [1, 2]. The use of ^68^Ga-labelled peptides (hereafter ^68^Ga-PET) is rapidly becoming the method of choice for SSTR imaging as its superiority over scintigraphy and/or SPECT/CT is obvious not only to the eye but also in terms of sensitivity, lower radiation exposure to the patient, and being a less time-consuming diagnostic procedure [3–5]. The fact that ^68^Ga-PET detects far more lesions than SSTR scintigraphy is especially noticeable for skeletal metastases and non-enlarged lymph node metastases. This affects treatment planning very directly as it may more correctly select patients for, for example, surgery or liver-specific therapy.

Apart from diagnosis and staging, ^111^In-octreotide imaging is also used for selection of NET patients for peptide receptor radionuclide therapy (PRRT) with ^177^Lu-DOTA-conjugated peptides, a treatment that has been proven both safe and efficient [6–10]. Selection is based on the so-called Krenning scale [11], a qualitative evaluation of tumour lesion uptake of ^111^In-octreotide relative to its uptake in healthy liver and spleen. If tumour uptake is higher than the uptake in the liver and/or spleen, it is predictive of response to PRRT [2, 10].

The possibility of replacing the qualitative image evaluation with a quantitative one is appealing for several reasons. By being able to quantify the activity uptake in tumour lesions, it may allow us to improve selection of patients for PRRT, and perhaps also for “cold” somatostatin analog treatment.

The possibility to accurately quantify the activity and volume of an uptake will depend on, for example, the size of the uptake, the level of activity in the surrounding background, and the method for segmentation. The segmentation can be performed using several different methods, e.g. delineating the volume of interest (VOI) on a CT image and transfer it to the SPECT or PET, threshold-based VOIs or by background-adapted threshold-based VOIs.

One important step towards the use of quantification in a clinical setting is to know the accuracy of the method used. The aim of this study was to investigate the differences in image quality and to investigate the accuracy of quantification of ^68^Ga-PET and ^111^In-SPECT images. This was performed by phantom measurements, and different methods for segmentation and quantification were evaluated.

## Methods

### Phantom and radionuclides

A NEMA IEC Body Phantom (Data Spectrum, Hillsborough, USA) containing a set of six fillable spheres with inner diameters of 10, 13, 17, 22, 28 and 37 mm (corresponding to volumes of 0.5, 1.2, 2.6, 5.6, 11.5 and 26.5 cm^3^) was used. A cylindrical lung insert with an outer diameter of 51 mm was placed in the centre of the phantom. Four different sphere-to-background activity ratios (SBRs) were used for both SPECT/CT and PET/CT imaging.

The SBRs were selected by an interim analysis of tumour-to-background ratios for five patient examinations each of ^111^In-Octreoscan™ and ^68^Ga-DOTATATE. The five patient exams for ^111^In-Octreoscan™ gave tumour-to-background ratios of 50, 3 and 1 for tumours in the lung, liver and spleen, respectively. The corresponding ratios for ^68^Ga-DOTATATE were 80, 4 and 1. Based on this, the phantom measurements were performed with no activity in the background (NB) to correspond to tumours in or close to the lung and the SBRs 5:1 and 2.5:1 to reflect tumours in the liver and 1.25:1 to reflect tumours in the spleen.

For the SPECT acquisitions, all six spheres were filled with ^111^In-Octreoscan™ from the same solution. Diethylene triamine pentaacetic acid (DTPA) (10 mMol/L) was added to the solution to prevent adhesion of the radionuclide to the phantom walls. For PET imaging, the spheres were filled with ^68^Ga obtained from a ^68^Ge/^68^Ga generator (IGG100, 50 mCi, Eckert & Ziegler). The same chelating agent (DTPA) as for the SPECT phantom was used for the PET phantom, and phosphate buffer was added to obtain a neutral pH value. To compare the results for ^68^Ga to a more commonly used radionuclide, the phantom was filled with ^18^F-FDG with a SBR of 5:1.

All activities for spheres and background were determined in calibrated dose calibrators (^111^In: CRC-15R, Capintec; ^68^Ga and ^18^F: CRC-15PET, Capintec) and corrected for decay till the start of the image acquisitions.

A cross calibration of the dose calibrator and the PET camera is performed four times per year and was controlled close in time to when the phantom acquisitions were performed.

### Image acquisition

The gamma camera measurements were performed using a dual-headed Philips Precedence SPECT/CT system equipped with a Medium Energy General Purpose (MEGP) collimator. The acquisitions were performed over 128 projections (180° and 64 projections per camera head) in a 64 × 64 matrix in step and shoot mode. The acquisition time was set to 28 s/angle. Two energy windows 171 keV ± 10% and 245 keV ± 10% were used for the imaging. Attenuation correction was performed using a low-dose CT acquisition. All SPECT images were reconstructed with Ordered Subsets Expectation Maximization (OSEM) with 16 iterations and 8 subsets and with the resolution recovery algorithm (Astonish). The reconstructed slice thickness was 9.3 mm and the pixel size was 9.3 mm/pixel.

The measured sensitivity factor for ^111^In for the SPECT images was determined from an acquisition of the NEMA IEC Body Phantom (without spheres and lung insert) filled with a uniform distribution of ^111^In. A cylindrical ROI was drawn centrally in the reconstructed images, and the value of mean counts per voxel was used as the measured sensitivity factor (cps/MBq voxel). The analysis of the SPECT images was performed at the gamma camera work station (Extended Brilliance Workspace, EBW, Philips).

All PET acquisitions (^68^Ga and ^18^F) were performed in list mode on a GE Discovery PET/CT 690. Images were acquired during 3 min in one bed position, with matrix size 192 × 192, 3.65 mm/pixel, and slice thickness 3.27 mm and time of flight (TOF). The images were reconstructed with OSEM, 3 iterations and 12 subsets, 5-mm post filter and a 3D model for the point spread function (SharpIR). CT-based attenuation correction was performed. The quantitative analysis of the PET images was performed at the PET/CT workstation (AW VolumeShare 5, GE).

Clinical acquisition and reconstruction protocols were used in this study, and the noise level in the background of the different phantom configurations was, for both the SPECT and the PET images, comparable to the noise level found in lesion-free liver of patients on both modalities.

### Qualitative analysis

Axial slices centered over the six spheres in the ^111^In-SPECT and the ^68^Ga-PET images were analysed visually and by line profiles drawn across the spheres with 17- and 37-mm diameter.

### Quantitative evaluation of the activity concentration

The activity concentration was quantified after segmentation of the spheres in both the ^111^In-SPECT and ^68^Ga-PET images. The activity concentration in the spheres was quantified by the maximum value in the VOI and the value of the mean activity concentration in the segmented sphere for the different methods.

The segmentation was performed by three methods:

1. Threshold-based VOIs
2. Threshold-based VOIs adapted to the background
3. CT-based ROIs

Threshold-based VOIs were drawn with threshold values from 10 to 90% in steps of 10. The threshold level that gave the best quantification of the activity concentration for the majority of the spheres for a given SBR was chosen to be presented in the “Results” section.

The background-adapted thresholds were calculated according to Eq. 1 [12]:

1 $$ {T}_{\mathrm{adapt}}=T\times \left({\mathrm{AC}}_{\mathrm{max}}-\mathrm{BG}\right)+\mathrm{BG} $$

where *T*_adapt_ is the background-adapted threshold, *T* is the uncorrected threshold value, AC_max_ is the measured maximum activity concentration in the VOI and BG is the mean value of the background. The value of BG was determined from three ROIs drawn in the background, between the spheres and the edge of the phantom. The VOIs based on the background-adapted threshold were drawn with threshold values from 10 to 90% in steps of 10. The results for the background-adapted threshold that gave the best quantification for the majority of the spheres for a given SBR were chosen to be presented in the “Results” section. The activity concentration in the ^18^F-PET images was quantified by the maximum value in the VOI and the mean activity concentration after segmentation of the spheres with CT-based ROIs.

The CT-based ROIs were drawn to delineate the inside border of the spheres in the CT image in the slice corresponding to the central slice of the spheres. The ROIs were copied into the ^111^In-SPECT, ^68^Ga-PET, and the ^18^F-PET images.

The maximum and mean number of counts for each sphere and each SBR was noted from the ^111^In-SPECT images and converted to activity concentration using the measured sensitivity factor. The maximum and mean activity concentration in kilobecquerel per millilitre was obtained from the ^68^Ga-PET and ^18^F-PET images.

The recovery coefficient (RC) was calculated as the ratio of the measured activity concentration and the true activity concentration. The recovery coefficients were calculated for each sphere, radionuclide, SBR and quantification method.

### Quantitative evaluation of the sphere volume

Threshold-based VOIs were drawn with threshold values from 10 to 90% in steps of 10. The threshold level which gave the most accurate estimation of volume (compared to the true sphere volume) was determined for ^111^In, and ^68^Ga for all spheres and background activity levels. The threshold level that gave the best quantification of each sphere volume for a given SBR was chosen to be presented in the “Results” section.

## Results

### Qualitative analysis

**Fig. 1:**
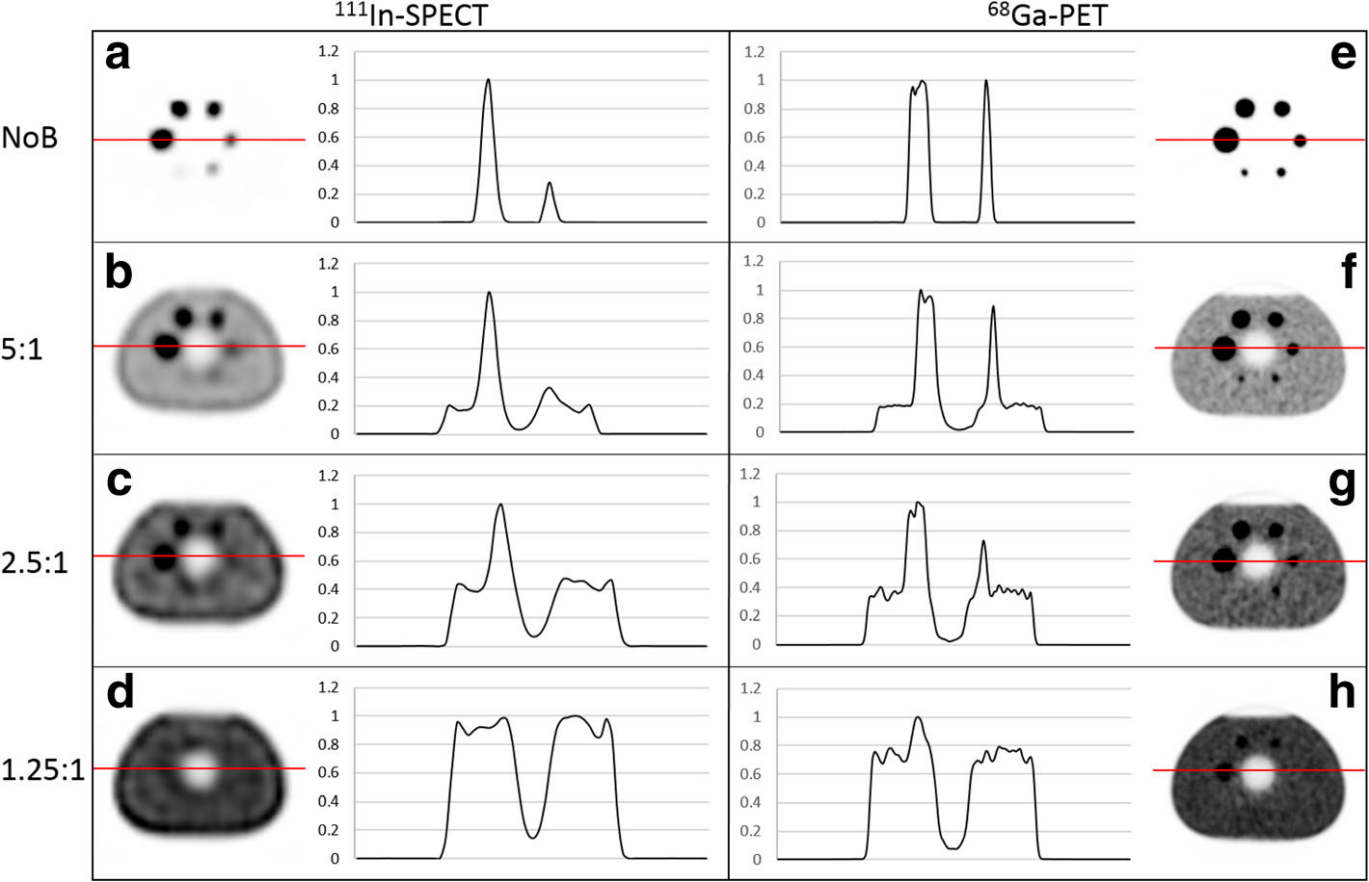
Line profiles over the spheres with an inner diameter of 37 and 17 mm in **a**–**d** show the spheres imaged with ^111^In-SPECT and **e**–**h** show the spheres imaged with ^68^Ga-PET

The ^111^In-SPECT and ^68^Ga-PET images of the phantom for the different SBRs are shown in Fig. 1. In the NB images, (Fig. 1a, e), the smallest sphere with an inner diameter of 10 mm was barely visible in the SPECT image whereas all spheres were visible in the PET image. In the images with a SBR of 5:1 (Fig. 1b, f), spheres equal to or larger than 17 mm were visible in the ^111^In-SPECT image, while all spheres were visible in the ^68^Ga-PET image. With a further increase of the background activity, SBR of 1.25:1 (Fig. 1d, h), no spheres were clearly seen in the SPECT image whereas spheres with inner diameters equal to and larger than 22 mm were visible in the PET image. To further compare the image quality of the ^111^In-SPECT and ^68^Ga-PET images, line profiles were drawn over the 37- and 17-mm spheres, in the SPECT images (Fig. 1a–d) and the PET images (Fig. 1e–h) for the different SBRs. The line profiles support the visual results of the axial slice images of the phantom. For example, for the highest background activity (SBR 1.25:1), the line profile shows no peaks at all related to the spheres in the SPECT images while the 37-mm sphere appears as a peak in the profile in the PET image.

### Quantification of the activity concentration

#### ^111^In-SPECT

**Fig. 2:**
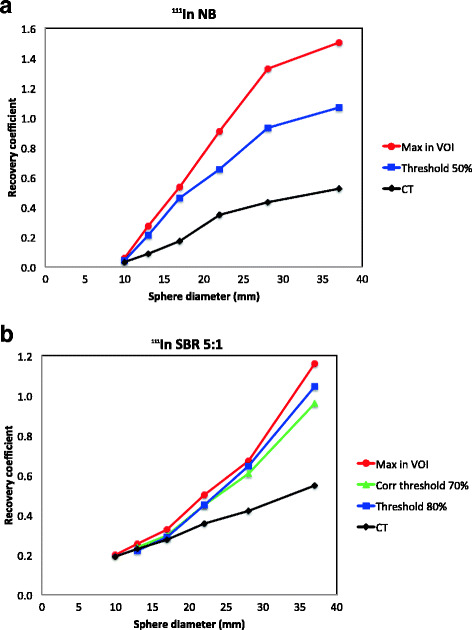
Recovery curves for the different quantification methods used in this study are shown for ^111^In-SPECT with no background activity (**a**) and for SBR 5:1 (**b**)

The accuracy of the quantification was dependent on the SBR, the size of the spheres and the quantification method used. In NB images, all spheres were possible to segment with a threshold-based VOI. The second largest sphere (28 mm) had a recovery coefficient of 0.93, the activity concentration of the largest sphere was overestimated and the activity concentration of the smaller spheres was underestimated (Fig. 2a). The recovery coefficients at SBR 5:1 are presented in Fig. 2b. The smallest sphere possible to segment using a threshold-based method was 13 mm. The curves in Fig. 2b show that the activity concentration was underestimated for all sphere sizes and all methods except for the largest sphere (37 mm) which had a recovery coefficient close to 1 for threshold-based (RC = 1.05), background-adapted (RC = 0.96) and maximum value in the VOI (RC = 1.16). Similar results were obtained for SBR 2.5:1. However, with the SBR of 2.5:1, the diameter of the smallest sphere possible to segment was 22 mm. The recovery coefficient for the largest sphere was close to 1 for threshold-based VOI at 90% threshold (RC = 1.01) and background-adapted VOI at 80% threshold (RC = 1.01). None of the spheres could be segmented with the threshold-based methods for the SBR of 1.25:1. The data for SBRs 2.5:1 and 1.25:1 are not shown. Mean counts in a CT-based ROI resulted in an underestimation of the activity concentration for all spheres and all SBRs (Fig. 2a, b). The smaller the size of the spheres, the larger the underestimation of the activity concentration was found to be. The recovery coefficient was also higher for higher background activities.

#### ^68^Ga-PET

**Fig. 3:**
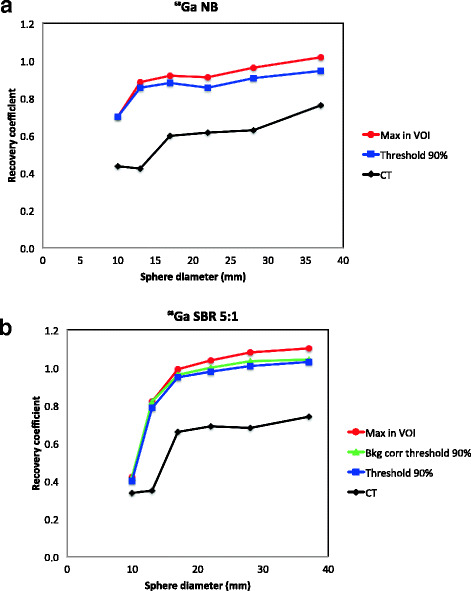
Recovery curves for the different quantification methods used in this study are shown for ^68^Ga-PET with no background activity in the phantom (**a**) and for SBR 5:1 (**b**)

The accuracy of the activity quantification was considerably higher, and more consistent, in ^68^Ga-PET than in ^111^In-SPECT images as illustrated in Fig. 3a. For smaller spheres, the accuracy of the quantification decreased as background activity level increased from NB to SBR 5:1.

It was possible to quantify the activity concentration within 10% of the true concentration using the maximum value in the VOI for the four largest spheres when no activity was present in the background (RC = 0.91–1.02). The recovery coefficient was 0.94 and 0.91 for the two largest spheres, respectively, using a threshold-based VOI. The RC was close to 1 for the four largest spheres with the threshold-based method (RC = 0.95–1.03) and the background-adapted method (RC = 0.96–1.04) for SBR 5:1 (Fig. 3b). The recovery curve for the maximum value in the VOI follow the curves for threshold-based and background-adapted VOIs, but with slightly higher values (RC = 0.99–1.10). The two smallest spheres were underestimated for all three methods (RC = 0.40–0.82). Similar results were obtained for SBR 2.5:1 using threshold-based VOIs at 90% threshold (RC = 1.00–1.09) and background-adapted VOIs at 80% threshold (RC = 0.98–1.07). Only the three largest spheres could be segmented using threshold-based and background-adapted VOIs for the highest background activity, SBR 1.25:1. The recovery was close to 1 for those three spheres for the maximum value in the VOI (RC = 1.03–1.06). The recovery coefficients for threshold-based at 90% and background-adapted at 70% VOIs were also equal to or close to 1 (RC = 0.97–1.0). The data for SBRs 2.5:1 and 1.25:1 are not shown. The same pattern as for the ^111^In-SPECT measurements was seen for ^68^Ga-PET for a CT-based ROI, i.e. that the activity concentration was underestimated for all spheres and SBRs (Fig. 3a, b).

**Fig. 4:**
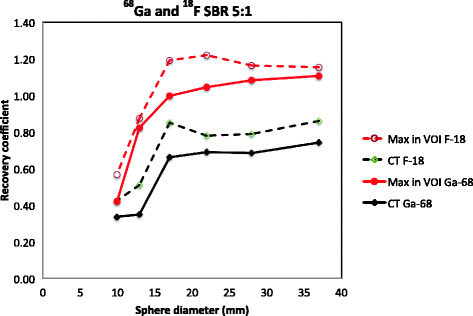
Recovery curves for maximum value in VOI and mean value in CT-based ROI for ^68^Ga and ^18^F for SBR 5:1

The recovery coefficient was approximately 15% lower for the measurements performed with ^68^Ga compared with ^18^F in the phantom as shown in Fig. 4.

### Quantitative evaluation of the sphere volume

No single threshold value was applicable to receive the correct volume for all SBRs and all sphere sizes neither for ^111^In-SPECT nor for ^68^Ga-PET.

#### ^111^In-SPECT

**Fig. 5:**
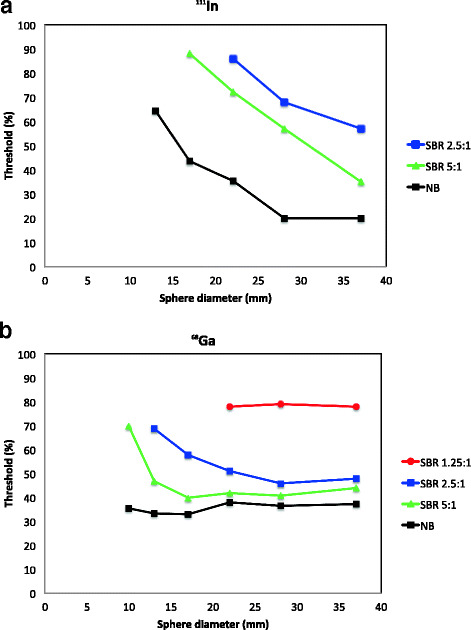
Threshold level giving the accurate volume for different sphere diameters and SBRs are shown for ^111^In-SPECT (**a**) and for ^68^Ga-PET (**b**). The data for SBR 1.25:1 was not possible to obtain for ^111^In-SPECT

The threshold values resulting in the most accurate volume estimation for the different SBRs in ^111^In-SPECT images are shown in Fig. 5a. The correct volumes of the two largest spheres could be determined with a threshold level of 20% with no activity in the background. Decreasing sphere volume required higher threshold levels to achieve accurate volume estimation. The volume was overestimated for all threshold levels for the smallest sphere (10-mm diameter) due to the large voxel size.

The volumes for the four largest spheres could be determined with an SBR of 5:1 and the three largest spheres for an SBR of 2.5:1. No sphere was visible with the highest background activity, SBR 1.25:1.

#### ^68^Ga-PET

Figure 5b shows the threshold levels needed to determine the correct sphere volume for ^68^Ga. All sphere volumes could be determined with SBR 5:1, SBR 2.5:1 and NB in the phantom. The three largest spheres were possible to quantify with SBR 1.25:1. With no background activity, threshold values of 36–38% resulted in volumes within 10% of the true volume for the three largest spheres. Corresponding threshold values were 41–44% for SBR 5:1 and 46–51% for SBR 2.5:1. The volume of smaller spheres was significantly overestimated using these thresholds, i.e. higher thresholds were needed for smaller spheres.

## Discussion

Based on the quantification methods investigated in this study, it was not possible to find a general method for the quantification of neither the activity concentration nor the volume in ^111^In-SPECT that gave a reasonably accurate result. This indicates that a qualitative evaluation of the images, e.g. Krenning scale [11], is the most useful approach for patient selection for therapy from ^111^In-SPECT images.

CT-based VOIs resulted in underestimation of the activity concentration in the ^68^Ga-PET images for all SBRs and sphere sizes due to partial volume effect and spill out of the signal from the activity in the sphere to the background area. For higher SBRs, this effect is partly balanced out by spill in of signal from the background to the sphere. However, the recovery coefficient was underestimated in all measurements in this study. The recovery curves presented in Fig. 4 were similar in shape. However, the curve for ^68^Ga was lower than that for ^18^F. The differences might be explained by different maximum positron energy and particle ranges. It might also be influenced by how the radionuclides are defined and by how the corrections for high-energy gamma photons are performed by the system.

The use of the threshold-based and the background-adapted methods in the ^68^Ga-PET images resulted in recovery coefficients close to 1 for medium-sized spheres (17–37-mm diameter). The threshold levels to achieve this were close to the maximum activity concentration (70–90%). The use of the maximum activity concentration in the VOIs resulted in slightly overestimated concentrations for the spheres surrounded by background activity. However, the use of the maximum activity concentration is a simple and fast way to estimate the activity concentration in ^68^Ga-PET images. The accuracy of the quantification of the activity concentration in spheres with a diameter of 17 mm and larger for ^68^Ga-PET with the maximum in VOI method is ± 20%.

Our results also indicate that, for ^68^Ga-PET, a threshold of about 40% (range 37–51%) for a threshold-based VOI can be applied to receive an approximate volume for larger spheres (22–37-mm diameter), except for the highest background level which corresponds to the situation in the spleen. The volume determined from the ^68^Ga-PET image can be determined with an accuracy of approximately ± 20% for spheres with a diameter of 22 mm and larger using this threshold-based VOI.

The quantification situation in a phantom is ideal, i.e. the activity is uniformly distributed within the spheres and the phantom does not move during the acquisition. Quantification of both activity concentration and tumour volume in a patient is hampered by a possibly non-uniform activity concentration in a tumour, non-spherical tumours and patient motion due to breathing and cardiac motion.

The voxel size in the clinical protocols used in this study is relatively large, a trade-off between sensitivity and spatial resolution. The volume of a voxel in the images in this study was 0.80 cm^3^ in SPECT and 0.044 cm^3^ in PET, which delimits the detection level of both the volume and the activity concentration of the smallest spheres in the NEMA phantom (0.52 and 1.15 cm^3^).

All reconstructions in this study included a 3D model for the point spread function. At high SBRs, the use of point spread function algorithms provides images with higher spatial resolution and low deviation in quantified mean activity concentration. However, the use of these algorithms causes Gibbs artifacts with an overestimation of the maximum activity concentration in the spheres [13].

An accuracy level of ± 20% in either activity concentration or tumour volume could presumably be accurate enough to be used for patient selection for therapy and/or for treatment evaluation. The clinically relevant criteria to be applied for this need to be investigated. The examples of selection criteria for therapy that could be investigated are the ratio of the activity concentration in tumours larger than 2 cm to the activity concentration in normal liver tissue, the absolute activity uptake in a tumour, or combinations of those. Criteria to investigate for treatment evaluation could be the viable tumour volume from the PET as a measure for treatment evaluation. Another alternative to investigate could be the absolute activity uptake in a tumour.

## Conclusions

Our results firmly support what has already been experienced, but not systematically analysed, in clinical practice––^68^Ga-PET imaging is superior to ^111^In-octreotide scintigraphy in several ways: a higher detection rate of tumour lesions, detection of smaller lesions and the possibility to quantify the activity in a lesion with reasonable accuracy. All these aspects are of importance to the clinician as these affect treatment choices and may aid in the prediction of treatment efficacy.

Based on the quantification methods investigated in this study, no quantification method gave a reasonably correct result neither for the activity concentration nor for the volume in ^111^In-SPECT. This indicates that a qualitative evaluation of the images, e.g. Krenning scale, is the most useful approach for ^111^In-SPECT.

For the ^68^Ga measurements, all spheres were visible in the images except at background levels corresponding to activity uptake of ^68^Ga-DOTATATE in the spleen where the spheres 22 mm and larger could be seen. The activity concentration could be determined with acceptable accuracy for spheres equal to or greater than 17 mm by using the maximum activity concentration in the VOI. For low background activity corresponding to uptake of ^68^Ga-DOTATATE in, e.g. the lungs, a threshold of around 40% gave a value of the sphere volume with acceptable accuracy.

## Abbreviations

CT: Computed tomography
DTPA: Diethylene triamine pentaacetic acid
MEGP: Medium Energy General Purpose
NB: No background activity
NET: Neuroendocrine tumour
OSEM: Ordered Subsets Expectation Maximization
PET: Positron emission tomography
PRRT: Peptide receptor radionuclide therapy
RC: Recovery coefficient
ROI: Region of interest
SBR: Sphere-to-background activity ratio
SPECT: Single-photon emission computed tomography
SSTR: Somatostatin receptor
VOI: Volume of interest
