# Correction to: Quantitative analysis of phantom studies of ^111^In and ^68^Ga imaging of neuroendocrine tumours

**DOI:** 10.1186/s40658-022-00502-x

**Published:** 2022-10-31

**Authors:** L. Jönsson, A. Stenvall, E. Mattsson, E. Larsson, A. Sundlöv, T. Ohlsson, C. Hindorf

**Affiliations:** 1 Department of Medical Radiation Physics, Clinical Sciences, Lund University, Lund, Sweden; 2 Department of Hospital Physics, County Hospital Ryhov, Jönköping, Sweden; 3 Department of Radiation Physics, Skåne University Hospital, Lund, Sweden; 4 Department of Oncology and Pathology, Clinical Sciences Lund University, Lund, Sweden

## Correction to: *EJNMMI Phys* 5, 5 (2018). https://doi.org/10.1186/s40658-018-0204-0

Following publication of the original article [1], it was reported that Eq. 1 was erroneously published as:

1 $$T_{adapt} = T \times \left( {AC_{max} - BG} \right) + BG$$

This equation should read as follows:

1 $$T_{{{\text{adapt}}}} = \frac{{T \times \left( {{\text{AC}}_{\max } - {\text{BG}}} \right) + {\text{BG}}}}{{{\text{AC}}_{\max } }}$$

The numerical values presented in the paper are calculated according to this corrected equation.
